# Recent trends in stem cell therapy for premature ovarian insufficiency and its therapeutic potential: a review

**DOI:** 10.1186/s13048-020-00671-2

**Published:** 2020-06-23

**Authors:** Jeeyoon Na, Gi Jin Kim

**Affiliations:** 1 Department of Biology, College of Arts & Sciences, University of Pennsylvania, Philadelphia, PA 19104 USA; 2 Department of Biomedical Science, CHA University, Seongnam, 13488 Republic of Korea

**Keywords:** Mesenchymal stem cell, Ovarian dysfunction, Animal model, Therapeutic effect

## Abstract

Stem cell therapy is attracting attention in the field of regenerative medicine and is advancing rapidly. Many recent studies have applied stem cell therapy to treat reproductive system diseases; however, data are not yet available as to whether this therapy shows enhanced therapeutic effects. This paper analyzes recent preclinical studies on stem cell therapy for ovarian dysfunction in several types of animal models. Several clinical trials and pending projects are also discussed. This review will provide a background for developing stem cell therapies to enhance ovarian function.

## Introduction

Stem cells are unique in that they have the ability to self-renew and differentiate into specific tissues according to the surrounding environment and signals. Stem cells can be roughly divided into pluripotent and multipotent stem cells according to their differentiation potential [1]. Pluripotent stem cells can differentiate into any cells derived from the endoderm, mesoderm and ectoderm, whereas embryonic stem cells (ESCs) are the most pluripotent of all stem cells; they are derived from the inner cell mass of the blastocyst and possess unlimited ability to differentiate into various cell types [2]. Despite their unlimited potency, ESCs are not frequently manipulated in research because of ethical reasons and the cancer risk, such as the risk of teratoma formation. The derivation of ESCs is often considered unethical by those who oppose the disruption of the embryo, since the process of isolating ESCs involves disrupting the blastocyst [3]. Considering such ethical concerns, there are alternatives of using ESCs, such as induced pluripotent stem cells (iPSCs) and mesenchymal stem cells (MSCs), which is mostly used in research. As an alternative pluripotent stem cell source, iPSCs are applied to exploit the advantageous potency of ESCs. iPSCs are artificial stem cells made by reprogramming specialized cells such as fibroblasts with limited potency. In this way, multipotent cells can be used without any ethical concerns [3]. Mesenchymal stem cells (MSCs) are multipotent adult stem cells. Although they are derived from the mesoderm only, they are widely used in research and therapeutics because of their ability to differentiate down multiple lineages of tissues/cells, such as chondrocytes, osteocytes, adipocytes, hepatocytes, and even neurons [4]. MSCs can be isolated from various sources, including bone marrow, fat tissue, amniotic fluid, umbilical cord, placenta and skin [5]. The advantages of MSCs are that they do not raise any ethical concerns, they have low immunogenicity and limited immunomodulatory function, and they undergo chemotaxis in wound healing. However, the self-renewal potential is limited by donor age and the invasive collection methods, including bone marrow aspiration and liposuction [6].

Many scientists have characterized several stem cells and evaluated their therapeutic effects in various animal models of degenerative diseases [7]. Based on the considerable data on the feasibility of stem cells for various degenerative diseases, clinical trials are ongoing to develop new stem cell-based treatment strategies in regenerative medicine [www.clinicaltrials.gov]. However, there are few reports on the effects of stem cells in reproductive diseases. In reproductive medicine, ovarian function is key in the health of both young and old women [8].

In particular, premature ovarian insufficiency (POI), which was previously referred as premature ovarian failure, defines a state of female hypogonadism which causes a loss of ovarian function before the age of 40 years [9]. POI can be caused by side effects of anticancer chemotherapy or by surgical means. This condition can be diagnosed based on a decrease in the number of follicles, abnormalities in the menstrual cycle, and infertility. The gold-standard diagnostic is a change in the levels of the main ovarian hormones [10]. For this reason, several animal models have been generated to test the applicability of stem cell therapies in treating infertility. Since many patients suffer from POI as a side effect of chemotherapy, chemically induced ovarian failure is commonly used in animal models. In addition, naturally aged (NOA) models, models in which one ovary is surgically removed, and knockout (KO) mice are used to study ovarian damage and the effect of stem cells. However, the therapeutic effects of stem cells are still controversial because of variation among animal models and physical differences between model animals and humans [11].

Therefore, we analyzed recent preclinical studies of stem cell therapy to treat ovarian dysfunction in several animal models. Additionally, we discuss several clinical trials and pending projects and provide the background for developing stem cell therapies to enhance ovarian function.

## Main body

### Premature ovarian insufficiency

Basically, POI is defined as ovarian dysfunction, including the early termination of ovarian function before the age of 40. POI can be diagnosed by a decrease in the number of follicles, abnormalities in the menstrual cycle, and infertility. The gold-standard diagnostic is the change in the levels of the main ovarian hormones (low estradiol (E2), follicle-stimulating hormone (FSH) ≤40 mIU/ml) [1]. Generally, ovarian function is controlled by the hypothalamic-pituitary-ovarian axis [12]. Gonadotropin-releasing hormone (GnRH) released from the hypothalamus prompts the anterior pituitary gland to secrete FSH and luteinizing hormone (LH), which act on the ovary. This pathway is negatively controlled by progesterone. FSH and LH are the main ovarian hormones that regulate folliculogenesis. Additionally, anti-Müllerian hormone (AMH) is secreted by granulosa cells (GCs) in the preantral follicle stage and during the early development of antral follicles. Therefore, AMH and E2 have negative feedback effects on follicular development and affect the level of FSH. When GCs are damaged by chemotherapy or other external stimuli, E2 and AMH levels fall, causing FSH to increase and leading to exhaustion of the follicular pool. The decreases in E2 and AMH and increase in FSH secretion are used to detect POI [13].

Today, the most efficient treatment for women with POI is hormone therapy. However, hormone replacement therapy may do more harm than good for many women because of its side effects (e.g., high cancer risk) [13]. Therefore, many scientists have attempted to develop alternative therapeutics using stem cell-based strategies.

### Animal models of ovarian dysfunction

Generally, mouse and rat models of POI are chemically induced by busulfan, cyclophosphamide, ZP3, etc. Cyclophosphamide and busulfan are often combined (CTX) to induce POI upon intraperitoneal (i.p.) or tail vein (intravenous, i.v.) injection [14]. Another way to study the mechanisms of premature termination of ovarian function is with naturally aged model (NOA) because they both show early termination of menstruation and lack the ability to generate follicles [15]. Thus, NOA model is appropriate to understand how stem cell therapies affect dysfunctional ovaries. These models are generated using mice or rats aged 12–14 months, and ovarian failure is confirmed by vaginal smears [16].

**Fig. 1:**
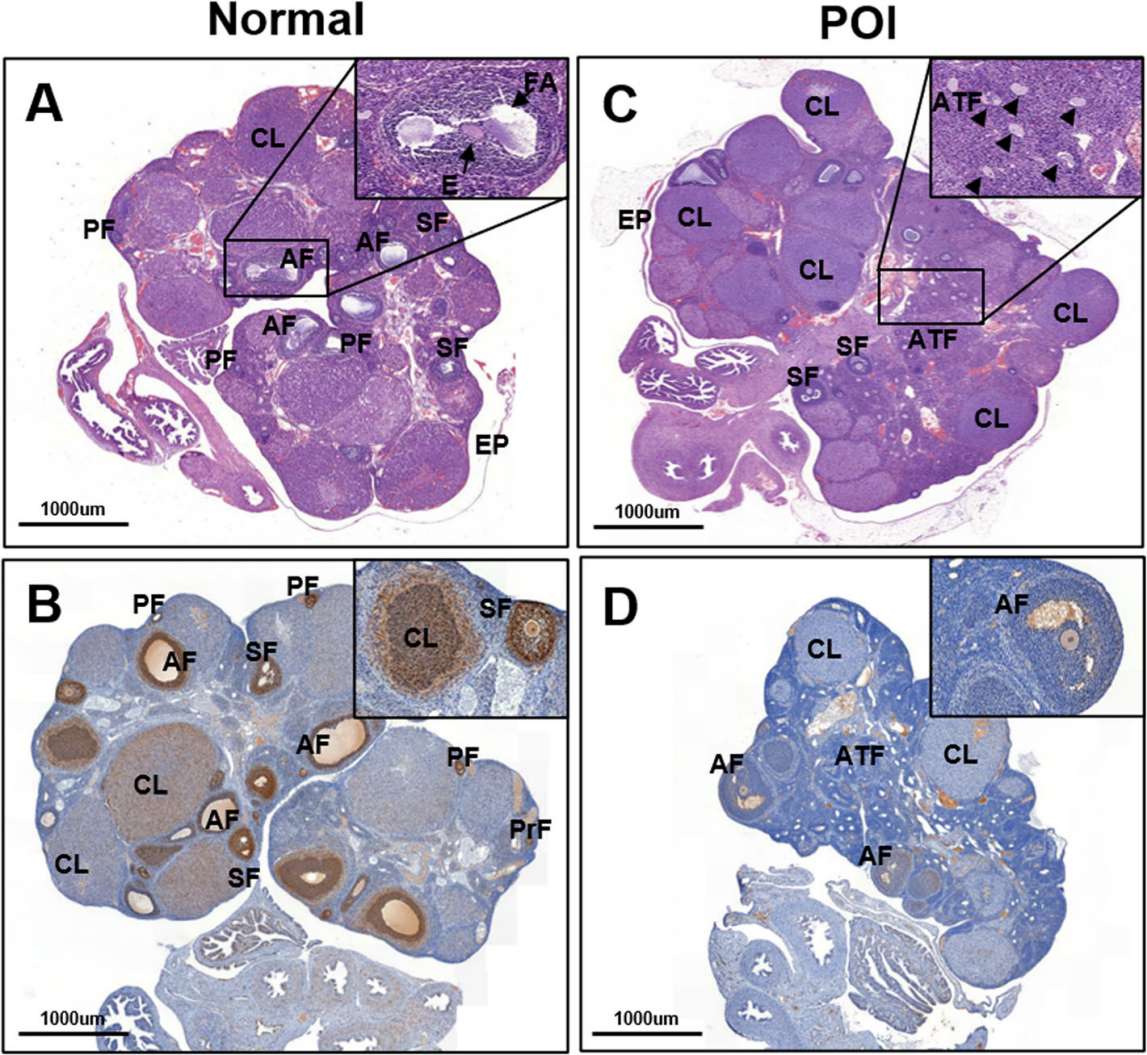
The morphological changes of ovary tissue by H&E and PCNA staining. Light microscopic examination of ovaries. H&E. (**a-b**) Normal ovary. (**a**) The ovary showing numerous mature follicles. (**b**) The PCNA expression in ovary is remarkably increased by immunohistochemistry (IHC). (**c-d**) POI ovary. (**c**) The Atretic follicles (arrowhead; ATF) are highly increased in POI ovary. (**d**) The PCNA expression in ovary is extremely decreased by IHC. Interestingly, PCNA expression is not detected in antral follicle of POI ovary. (Low magnification: ×15, High magnification: ×100). Primordial follicle (PrF), Primary follicle (PF), Secondary follicle (SF), Antral follicle (AF), Corpus luteum (CL), Follicular antrum (FA), Egg (E), Atretic follicle (ATF) and Surface epithelium (EP)

Since one of the causes of the premature termination of ovarian function is surgical procedures that cause ovarian injury, ovariectomized (OVX) models are also studied [17]. OVX models are used to study how stem cell therapy can improve ovarian function when one whole ovary or one-half of each ovary (1/2 OVX) is removed (Fig. 1) [18]. Finally, mice with specific genes knocked out are commonly used to identify the genes responsible for POI [19]. Follitropin receptor KO (FORKO) mice are a model used to study the genes related to the development of POI. A previous study showed that intravenously injected bone marrow-derived mesenchymal stem cells (BMMSCs) reached the ovaries of FORKO mice, differentiated and expressed the FSH receptor, thereby regulating FSH levels and estrogen production and promoting activate folliculogenesis [20]. Additionally, Bax KO mice show improved ovarian function, which typically decreases because of age-related issues. The proapoptotic gene Bax is related to aging. In Bax KO mice, the increase in ovarian function was more evident than the increase in lifespan [21].

However, representative animal models of POI have yielded controversial results because of differences in POI mechanisms, different experimental conditions, individual variation among animal models, and physical differences between model animals and humans [22].

### Sources of stem cells

ESCs can be isolated from the inner cell mass of the blastocyst; these are the only stem cells that have an unlimited potential to differentiate into any cell type. However, there are many ethical concerns with the use of these cells since their isolation requires destroying the fetus at the very early stage, and thus, they are not usually used in research. Instead, iPSCs are used when research requires multipotency, but most studies rely on multipotent stem cells.

There are multiple sources of multipotent stem cells in both adults and fetuses. Some are autologous, meaning that they can be transplanted into the donor, and some are allogeneic, meaning that they can be transplanted into patients other than the donor [23]. Stem cells that can be isolated from adults include BMMSCs, adipose-derived mesenchymal stem cells (ADSCs) and peripheral blood mononuclear cells (PBMCs). Bone marrow is one of the most common sources of stem cells; it can be isolated from any adult and transplanted in both an autologous and allogeneic manner [24]. These stem cells are relatively easy to isolate compared to other stem cells and are thus safer.

Some stem cells can be isolated when a woman is pregnant or after she gives birth. Several types of stem cells are derived from the amniotic fluid. Human amniotic epithelial cells (hAECs) and amniotic fluid stem cells (AFSCs) are often referred together as human amniotic stem cells and are isolated from the amniotic fluid or membrane [25]. Some of these cells are also called amnion-derived mesenchymal stem cells (hAD-MSCs). After the child is born, the umbilical cord is a source of MSCs; umbilical cord mesenchymal stem cells (UCMSCs) and umbilical cord vein mesenchymal stem cells (hUCV-MSCs) show very low immunogenicity and can be used for allogeneic treatments. The placenta is another source of MSCs; human chorionic plate-derived mesenchymal stem cells (CP-MSCs), also known as human placenta-derived mesenchymal stem cells (hPMSCs), are commonly used in studies on the restoration of ovarian function [26].

Several stem cells are isolated from women only, including oogonial stem cells (OSCs) and menstrual blood-derived stromal cells (MenSCs), which have recently been used in multiple studies because of their relative convenience in isolation [27]. Several recent studies have shown that stem cells activate related cells and participate in paracrine and autocrine signaling pathways. Many experiments have shown that factors secreted by stem cells have restorative effects. The secretomes of BMSCs, exosomes of ADSCs extracted from human GCs, and hAD-MSCs are known to restore ovarian function in chemically induced POI models. However, these released factors are not the only causes of increased ovarian function, since conditioned media from stem cell cultures are not as efficient as the stem cells themselves. Further research is needed to elucidate the detailed pathway by which these MSCs interact with the ovary [28, 29].

### Potential therapeutic effects

Scientists recently reported that stem cell transplantation improves ovarian function by increasing the expression of related hormones and by preventing GC apoptosis, which suggests a therapeutic potential [30]. As shown in Table 1, the therapeutic effects of stem cells can be measured by several factors, such as folliculogenesis, the GC apoptosis rate, vascular formation, the pregnancy rate and the regulation of hormone levels (Table 1) [42]. Folliculogenesis can be promoted by the transplantation of stem cells, which can accelerate oogenesis or prepare an adequate environment. ADSCs are known to enhance the rigidity of theca cells and GCs, resulting in an optimal environment for folliculogenesis [41]. PBMC populations include various multipotent progenitor cells, such as stem cells, and have the ability to restore organ function and regenerate tissues. The combination of platelet-rich plasma (PRP) and PBMCs has a synergistic effect on restoring folliculogenesis when transplanted into the CTX-induced premature ovarian insufficiency (POI) model [46]. In addition, human endothelial progenitor cell (hEPC) transplantation attenuates the effects of aging on reproductive health, restores the capacity for embryo development, and re-establishes the regulation of inflammation, apoptosis and ER stress [33].

**Table 1:** Preclinical study of stem cell therapy on POF

Category	Stem cell type	Summary	Transplantation Details	Reference
Chemically induced model	UCMSC	■ UCMSCs restore the ovarian function after paclitaxel injection through a direct triggering effect on the ovarian epithelium and/or indirect enrichment of ovarian niche through regulating tissue expression of CK 8/18, TGF-ß and PCNA (epithelial tissue, growth factor etc)	injection of HCB-MSCs	[31]
		■ UCMSCs restore disturbed hormone secretion and folliculogenesis by reducing GC apoptosis. UCMSCs could reside in ovarian tissues and could survive for a comparatiely long time without obvious proliferation.	1 × 10^6^ UCMSCs intravenous injection	[32]
		■ UCMSC treatment restores ovarian function by increasing follicular number, decreasing FSH serum and increasing AMH serum	5 × 10^5^ UCMSCs injected via intraovarian route	[33]
		■ UCMSCs could reduce ovarian failure due to premature senescence caused by chemotherapy, and the NGF/TrkA signaling pathway was involved in the amelioration of POF.	5 × 10^6^ UCMSCs injected intravenously	[34]
		■ Serum levels of E2, LH, VEGF in hAMSC and UCMSC transplanted groups were greater and FSH level was lower. Transplantation restored damaged ovarian function.	1 × 10^6^ UCMSCs and h-AMSCs intraovarian injection	[24]
	BMMSC	■ Estradiol level dropped and FSH level rised 21 days after MSC therapy Damaged ovaries after chemotherapy regain functions after transplantation.	0.5 × 10^6^ BMMSCs injected through the tail vein	[23]
		■ BM-MSC transplantation produces healthier ovarian follicles and less apoptosis of ovarian cells	1 × 10^6^ BM-MSCs injected via tail IP	[35]
		■ The significant reduction of atretic follicle and significant increase of antral follicle and secondary follicle were observed in ovaries of BMSCs-treated mouse. mRNA expression levels of Nano3, Nobox, Lhx8 increased.	2 × 10^6^ BMMSCs injected via tail vein	[36]
		■ Restore ovarian hormone production (FSH, AMH), reactivate folliculogenesis	5 × 10^5^ BMSCs injected via intraovarian route	[13]
		■ BMMSC treatment resulted in higher numbers of preovutory follicles, metaphase II oocytes, 2-cell embryos, promoted ovarian vascularization, reduced apoptosis	1 × 10^6^ BMMSCs IP injection	[37]
	FGSCs	■ Transplanted FGSCs restored function of premature ovarian failure and generated offspring in mice model.	1 × 10^4^ FGSCs injected	[30]
	MenSCs	■ MenSC reduce apoptosis in granulosa cells, reduce fibrosis of ovarian interstitium, increase follicular numbers, reparative effects on damaged ovaries by secreting FGF2	2x10^6^MenSCs	[26]
		■ MenSCs regulate normal follicle development, estrous cycle, reduce apoptosis in ovaries and activate ovarian transcriptional expression in ECM-dependent FAK/AKT signaling pathway	1x10^6^MenSCs IP injection	[6]
	CP-MSCs	■ Restored serum hormone level and ovarian function	2 × 10^6^ chorionic plate-derived MSCs injected via tail vein	[38]
	hPMSC	■ Inhibits granulosa cell apoptosis and follicular atresia by upregulating expression of AMH and FSHR in granulosa cells	1x10^6^hPMSCs injected via tail vein	[25]
		■ hPMSC transplantation induces ZP3 immunization and restores ovarian function associated with PI3k/Akt signal pathway	1 × 10^6^hPMSCs IP injection	[39]
		■ hPMSC transplantation reduces apoptosis of GC by regulating the expression of IRE1-alpha pathway of ER stress in ovaries	1 × 10^6^ hPMSCs injected via tail vein	[40]
		■ PBMCs combined with PRP restore ovarian function by increasing ovarian neovascularization, folliculogenesis and reducing GC apoptosis	4 × 10^6^ PBMCs IP injection	[41]
	ADSCs	■ Exosomes derived from hADSCs improve ovarian function by improving follicle numbers and inhibiting apoptosis rate	1x10^6^hADSCs injected via intraovarian route	[27]
		■ Adipose tissue-derived MSCs improve follicular count, AMH and E2 levels, and related gene expressions (CXCL12, BMP-4, TGF-β, IGF-1)	1 × 10^6^AT-MSCs IP injection	[42]
		■ ADSCs transplantation on collagen scaffolds improved fertility of rats with ovarian damage.	2 × 10^6^ ADSCs injected via intraovarian route	[43]
	hAD-MSCs	■ hAD-MSCs inhibit chemotherapy-induced GC apoptosis, promote angiogenesis, regulate follicular development by upregulating Bcl-2 expression.	4x10^6^hADMSCs IP injection	[28]
	BMMSC, OSC	■ The in-vivo transplantation of OSCs can be more effective protectors than BMMSCs for follicle maturation after chemotherapy. Unfortunately, the source of OSSCs is still a limitation for clinical applications.	2 × 10^6^ BMMSCs and OSSCs injected intraperitoneally	[44]
Naturally aged model	hUCMSC	■ hUCMSC increase E2 and AMH, decrease FSH. Improves follicle number and expression of HGF, VEGF, IGF-1	1x10^6^hUCMSCs injected via tail vein	[14]
	hAMSC, hAEC	■ hAMSCs are more effective in improving ovarian function than hAECs based on its telomerase activity, pluripotent marker expression levels, cytokine secretion.	Intraovarian injection	[45]
	hADSC	■ HGF and bFGF derived from hADSCs improved ovarian function during natural aging via reduction of oxidative stress by activating the SIRT1/FOXO1 signaling pathway.	Intraovarian injection	[15]
	BMSC	■ intra-ovarian injection of BMSCs changed the gene expression but did not recover granulosa cells or ovarian tissue.	1 × 10^7^ BMSCs intraovarian injection	[22]
	hEPC	■ hEPCs attenuates reproductive aging and dysfunction potentially via regulation of inflammation, apoptosis and ER stress.	5x10^4^hEPCs injected via tail vein	[46]
Genetic causes	AFSC	■ AFSC-derived exosomes contain miR-146a and miR-10a which inhibit apoptosis in damaged GCs and prevent ovarian follicles from atresia	Direct injection	[47]
	miR-21-MSCs	■ miR-21 overexpression in MSC decreases apoptosis, downregulate PTEN and PDCD4, icrease E2, decrease FSH.	1 × 106 MSCs injected into the bilateral ovaries	[48]
	hAEC exosomes	■ hAEC exosomes increased number of follicles, inhibited GCs apoptosis by transferring miR-1246 which targets the apoptosis pathway.	1.6 × 10^9^ particles injected via tail vein	[49]
Surgical model	ADSCs	■ Adipose derived mesenchymal stem cells can prevent the destructive effects of ischemia reperfusion injury on grafted ovaries through reducing oxidative stress and inflammation leading to improvement in the follicular pool and the endocrine function of the autografted ovaries.	5 × 10^4^ ADSCs injected via intraovarian route	[55]
	PD-MSC	■ Spheroid-cultured PD-MSC transplantation increases estrogen and folliculogenesis-related gene expressions in Ovx rats	1 × 10^6^ PD-MSC harvested, 100,000 cells directly injected into ovary	[50]

*UCMSC* umbilical cord mesenchymal stem cell, *AFSC* Amniotic Fluid Stem Cell, *CP-MSCs* Chorionic plate-derived mesenchymal stem cells, *BMMSC* bone marrow derived mesenchymal stem cells, *FGSC* Female germline stem cell, *MenSC* Menstrual blood-derived stem cell, *hPMSC* human placenta-derived mesenchymal stem cells, *ADSC* adipose derived mesenchymal stem cell, *hAD-MSC* human amnion derived mesenchymal stem cell, *OSC* ovarian stem cell, *hAMSC* human amniotic mesenchymal stromal cells, *hAEC* human amniotic epithelial cell, *hEPC* human endothelial progenitor cell, *PD-MSC* placenta derived mesenchymal stem cell

UCMSCs interact with steroidal hormones by decreasing FSH levels and increasing AMH levels [32]. Treatment with UCMSCs recovers serum hormone levels, and although these levels are not completely within the normal range, they are better than those in the POI group. Ovarian weight and follicle number also increased, leading to the full recovery of ovarian function and the ability to become pregnant [50]. hAD-MSCs and hCP-MSCs are also known to regulate the levels of hormone such as E2 and AMH and to restore follicular growth [44]. Additionally, in a rat OVX model, the engraftment efficiency of spheroid CP-MSCs into residual ovarian tissue is higher than that of naive CP-MSCs [44]. Ovarian stromal stem cells (OSSCs) have been isolated from fetal ovarian tissue [40]. Despite the reduced accessibility compared to BMMSCs, OSSCs are better at restoring ovarian function by activating quiescent local stem cells via paracrine and autocrine signaling [40].

Another therapeutic mechanism to prevent ovarian failure is to inhibit GC apoptosis. hPMSCs can inhibit GC apoptosis by modulating stress levels controlled by the IRE1α pathway [35]. One of the newest therapeutic approaches is to use stem cells extracted from human menstrual blood. Human MenSCs began to be used in stem cell therapy mainly because any invasive methods are not necessary to extract these cells. Jalalie et al. reported the successful engraftment of MenSCs extracted from women aged 25 ~ 35 in a CTX-induced POI mouse model; these implanted cells inhibited apoptosis and cell cycle arrest through the ECM-dependent FAK-AKT signaling pathway. Further studies are necessary to identify possible differences in MenSCs between normal donors and POI patients [6].

Genetic components are the leading causes of ovarian dysfunction, and it is thus important to identify the responsible genes or miRNAs. BMMSC transplantation in a CTX-induced POI model inhibited the apoptosis of ovarian cells by reducing the levels of apoptosis-related genes such as Bax, p53, caspase-3 and Bcl2 [51]. The efficiency of BMMSC transplantation and proliferation after engraftment can be upregulated by heat shock pretreatment [47].

Several studies have shown the paracrine activity of stem cells by identifying exosomal miRNAs from stem cells in fetal tissues and studying their target genes. Exosomes derived from AFSCs contain miR-146a and miR-10a, which both target genes that induce GC apoptosis [49]. miR-146a or miR-10a KO mice did not show many differences, but deleting both genes decreased the therapeutic effects of AFSC transplantation. Additionally, miR-1246 was found in exosomes derived from hAECs, which can be separated from the amniotic membrane. hAEC-derived exosomes contained miR-1246, which helps protect follicles from toxic chemotherapy by regulating the apoptosis rate of GCs [48]. miRNAs are also used to enhance the outcome of MSC transplantation by regulating cell viability. In particular, miR-21 is known to inhibit GC apoptosis. MSCs transfected with a lentiviral vector to overexpress miR-21 showed increased viability, and the ovulation rate increased after transplantation of these cells in rats [52].

The most clear and obvious study is to measure the pregnancy rate after stem cell treatment. The recovery of ovarian function was proven in naturally breeding POI models that underwent BMMSC transplantation prior to mating with healthy male animals. This study reported a 100% pregnancy rate, indicating that these POI mice became pregnant at least once after BMMSC transplantation. However, oocytes extracted from these POI mice after stem cell transplantation were difficult to fertilize with healthy sperm in vitro. This finding highlights the current limitations in applying this stem cell therapy to IVF, and further research on this topic is needed [14].

### Clinical trials

There are several ongoing clinical trials in many countries registered in the NIH clinical trial database (*www.clinicaltrials.gov*). Recent clinical results indicate that stem cell transplantation enhances ovarian function as evidenced by resumed menstruation, regulated hormone levels and, in rare cases, the ability to become pregnant. To accurately analyze the therapeutic effects of stem cells, it is important to recruit the appropriate participants. Among the clinical studies mentioned in Table 2, there were some similarities in the inclusion and exclusion criteria. Most studies included patients with FSH levels greater than 20 or 25, aged younger than 40 years, and with a normal karyotype and a POI diagnosis. Patients with autoimmune diseases, breast cancer, ovarian cancer, secondary ovarian failure due to hypothalamic issues, infectious diseases including HIV or hepatitis, and gene defects were excluded from the study.

**Table 2:** Clinical study of stem cell therapy on POF

Status	Phase	Stem cell type	Sponsor	Clinical Trial Number
Completed	1,2	OCT4 marker measured	Al-Azhar University	NCT02151890
		hUCMSC and hCBMNC transplantation	Shenzhen Beike Bio-Technology Co., Ltd	NCT01742533
		autologous MSCs injection	El-Rayadh Fertility Centre	NCT02043743
		autologous MSCs treatment + OCT4 marker measured	Sayed Bakry	NCT02062931
Recruiting	–	BMSC treatment directly to ovary	University of Illinois at Chicago	NCT02696889
	1	embryonic stem cell derived MSC-like cell transplantation directly into bilateral ovaries	Chinese Academy of Sciences	NCT03877471
	1,2	VSELs from the patient’s peripheral bloodinjected in bilateral oviducts + hormone and menstrual conditions measured	Fuda Cancer Hospital, Guangzhou	NCT03985462
	–	Derivation of hESC lines	Hadassah Medical Organization	NCT00353197
Active	2	hUCMSC treatment	Sclnow Biotechnology Co., Ltd.	NCT03816852
	1,2	intraovarian transplantation of autologous BMSCs & MSCs.	Stem Cells Arabia	NCT03069209

*hUCMSC* human umbilical cord mesenchymal stem cell, *hCBMNC* human cord blood-mononuclear cells, *BMSC* bone marrow derived mesenchymal stem cells, *VSEL* very small embryonic-like stem cell, *hESC* human embryonic stem cell

A study conducted by M. Edessy evaluated ten POI patients based on the Edessy ovarian reserve score (EORS), which integrates AMH, FSH, and E2 levels with total antral follicle count and mean ovarian volume. The results showed that among 10 patients, 2 resumed menstruation 3 months after MSC transplantation, and one became pregnant (Table 2). Another study showed that UC-MSC transplantation with collagen scaffolds can increase the efficiency of transplantation by improving cell attachment and proliferation [43]. A clinical trial was designed with 2 treatment groups: one receiving UC-MSC transplantation and one receiving collagen/UC-MSC transplantation directly into their ovaries. The group that received collagen/UC-MSCs showed an improvement in ovarian function, and one patient became pregnant after treatment.

Among the 10 clinical trials in the NIH database, only 4 have been completed; the rest are ongoing, recruiting and/or active. Research subjects have been selected by strict standards. Most clinical studies have included POI patients with a normal karyotype and FSH levels greater than or equal to 20 IU/L. Most studies have excluded pregnant or lactating patients and those with autoimmune diseases, breast cancer, ovarian cancer, an abnormal karyotype, infectious diseases or other ovarian diseases such as endometriosis. Although there are several active ongoing clinical trials for POI using stem cell-based therapeutics, there are restrictions in each country regarding ethical and clinical concerns from the health-care community [39]. Potential of stem cell therapies in treating POI still needs further research and clinical results.

## Discussion

**Fig. 2:**
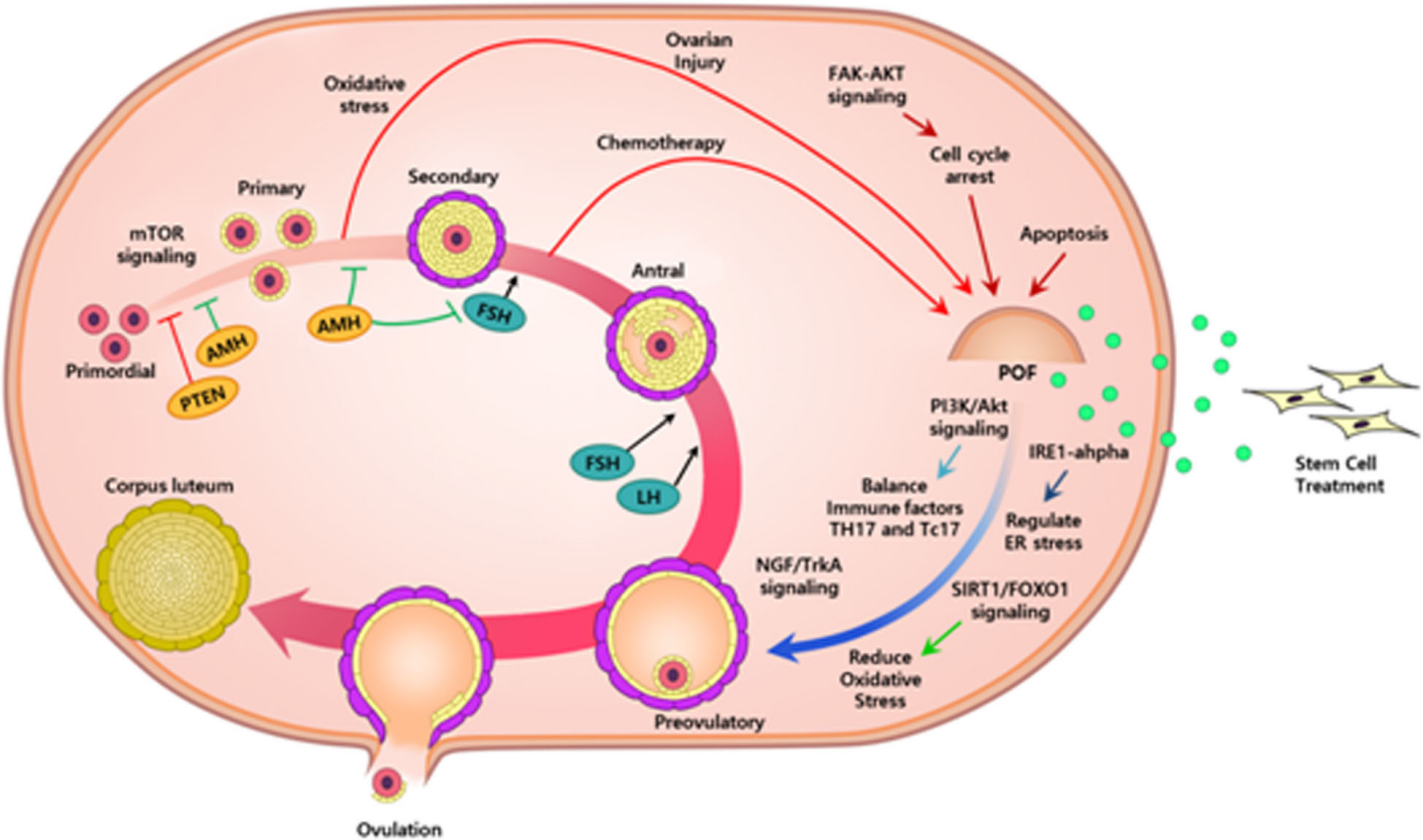
Summary on the therapeutic effect of mesenchymal cells derived from several sources on animal models with ovarian dysfunction

The therapeutic effects of stem cells have already been proven by various studies using animal models and clinical trials. It is obvious that stem cells can reinforce and revive ovarian function and therefore positively affect folliculogenesis, prevent GC apoptosis and regulate ovarian hormones (Fig. 2). However, there are some ethical and technical problems regarding the use of stem cells and stem cell therapies remain criminal in several countries. Although ethical concerns of using ESCs can be solved by using MSCs instead, there are some lingering safety concerns to be resolved in the process of extracting and transplanting stem cells for therapeutic usage [3]. MSCs derived from adipose cells, the placenta or the umbilical cord can be extracted using minimally invasive procedures that do not harm the donor. Direct transplantation into the patient can be invasive and potentially trigger side effects such as immune responses. These issues should be evaluated in additional in vivo studies and clinical trials.

In addition, there are limitations in designing animal models. First, it is difficult to produce the exact animal model and apply the results precisely to human patients. Differences in the immune system between animals and humans are one of the most important problems because they can affect the immunogenicity of transplanted stem cells. Thus, stem cells can not only have different effects when transplanted into humans but also elicit side effects such as autoimmune responses [11]. These limitations are evident in the results of clinical trials. Although mice and rats have shown an increase in folliculogenesis, a reduction in GC apoptosis and other positive effects related to the restoration of ovarian function, the success rate of clinical trials is not always 100%. More research is needed to design the perfect cell therapy for each patient and to reduce the risk of an immune response against transplanted allogeneic stem cells.

Second, there are variations among experimental animal models. POI is induced in animal models using various chemicals. Most studies have used busulfan, cyclophosphamide and ZP3, but the details, including the dose, timing of injection, and combination of chemicals, vary. The most common chemical regimen is 120 mg/kg cyclophosphamide, 12 mg/kg busulfan, or 50 mg/kg CTX. Furthermore, the chemical-induced ovarian damage can be spontaneously restored in an animal model, depending on the timing of cessation of chemical exposure. These variations in treatment conditions make it difficult to draw a conclusion about the efficacy of stem cell therapy in treating POI. Therefore, the details regarding various factors involved in the construction of an optimal animal model for POI are important.

Third, variations in the stem cell treatment dose must be considered. The optimal culture conditions of stem cells can differ according to the environment and source. Additionally, treatment designs differ according to the purpose of the research. For example, animal models differ based on whether the research intends to analyze the effect of stem cell therapy on POI, osteoporosis, or other kinds of dysfunction. Not all studies that claim the efficacy of stem cell therapy in restoring ovarian function intended to study POI. Complex aims have led to differences in the dose of transplanted stem cells, even within the same type of stem cell. For example, among various studies on transplanting AD-MSCs, cell numbers of 2 × 10^6^ and 4 × 10^6^ have been injected, even in the same animal model [37]. These variations can be found with any type of stem cell, including BMMSCs, MenSCs and PBMCs, as shown in Table 1 [36].

Additionally, the stem cell injection route can influence the outcome of stem cell transplantation. The various transplantation routes include the intraovarian route, i.v. route, i.p. route and lateral tail vein route. The transplantation route differs according to the purpose of the study. For example, studies on hPMSCs have used i.p. injection or tail vein (i.v.) injection, depending on the research purpose [31]. Studies on restoring ovarian function commonly inject stem cells directly into the ovaries via the intraovarian route, whereas studies considering the various effects of stem cell transplantation on more than one organ or tissue often use the i.p. or i.v. route in order to enable the spread of injected stem cells via the blood stream. These variations within and between animal models make it difficult to standardize the condition for efficient stem cell therapy in treating POI, and therefore, the results vary, even when using the same stem cells. Nevertheless, the results of individual studies have been positive, with the normalization of hormonal levels, restoration of mature follicle production, resumption of the menstrual cycle and, in rare cases, pregnancy [51]. Folliculogenesis is regarded to be as important as pregnancy rate when determining ovarian function [31]. Some studies have shown that the expression of folliculogenesis factors increases after stem cell treatment [45].

The results of follicles counts in each stage have indicated that the numbers of antral follicles and secondary follicles increase after stem cell treatment [45]. Engrafted stem cells interact with the surrounding environment and affect the levels of growth factors and immune factors [34, 53]. For instance, hAMSC transplantation increased the expression of multiple growth factors (hGC + EGF), which improved the proliferation rate and inhibited the apoptosis rate [54]. Moreover, these cells interfere with various signaling pathways to prevent apoptosis or oxidative stress [38]. Therefore, we can conclude that stem cell therapy has a positive effect in treating POI, and further research and clinical trials are needed to standardize the treatment details and determine a safe way to apply this technology in humans.

## Conclusion

It is evident that stem cell therapies have potential in treating POI. Stem cells and their exosomes, including content such as miRNAs, show positive effects in enhancing and restoring various aspects of ovarian function, such as folliculogenesis, the GC apoptosis rate, vascular formation, and genetic stability. Since stem cells are a proven new therapeutic strategy for the future cure of ovarian dysfunction, further studies are required to evaluate their therapeutic mechanism of action, to build a standard for quality control in clinical application, and to generate legal regulations to ensure safety.

## Data Availability

This manuscript is a review paper. So, “Not applicable”.
